# Trustworthy-Looking Face Meets Brown Eyes

**DOI:** 10.1371/journal.pone.0053285

**Published:** 2013-01-09

**Authors:** Karel Kleisner, Lenka Priplatova, Peter Frost, Jaroslav Flegr

**Affiliations:** 1 Department of Philosophy and History of Science, Charles University in Prague, Prague, Czech Republic; 2 Department of Anthropology, Université Laval, Quebec City, Canada; New York University, United States of America

## Abstract

We tested whether eye color influences perception of trustworthiness. Facial photographs of 40 female and 40 male students were rated for perceived trustworthiness. Eye color had a significant effect, the brown-eyed faces being perceived as more trustworthy than the blue-eyed ones. Geometric morphometrics, however, revealed significant correlations between eye color and face shape. Thus, face shape likewise had a significant effect on perceived trustworthiness but only for male faces, the effect for female faces not being significant. To determine whether perception of trustworthiness was being influenced primarily by eye color or by face shape, we recolored the eyes on the same male facial photos and repeated the test procedure. Eye color now had no effect on perceived trustworthiness. We concluded that although the brown-eyed faces were perceived as more trustworthy than the blue-eyed ones, it was not brown eye color *per se* that caused the stronger perception of trustworthiness but rather the facial features associated with brown eyes.

## Introduction

It has repeatedly been shown that facial appearance strongly affects various social interactions [1], [2]. Face perception is domain-specific, heritable, and independent of other cognitive abilities [3]. It should be highly adaptive in any social environment, especially for distinguishing a friend from a foe. In particular, primates show a highly developed capacity to recognize faces accurately even after the viewing position has been changed [4]. Humans are no exception.

Face perception provides information on trustworthiness, which in turn assists an individual’s social, economic, and reproductive success [5], [6]. During economic trust games, male participants with broader faces were more likely to exploit their counterparts’ trust than were male participants with narrower faces, and other players were more likely to entrust their money to males with longer and narrower faces [7]. In the human brain, the amygdala has been identified as the key structure for automatic evaluation of trustworthiness [5], [8]. Patients with bilateral amygdala damage cannot judge the trustworthiness of other persons appropriately [9]. Perceived trustworthiness also correlates with perceived happiness. Conversely, untrustworthy faces are considered to be angrier [10].

Most previous research has ignored the eyes as a facial feature that may influence personality assessment. Yet the eyes are highly conspicuous because the color of the iris contrasts with the whiteness of the sclera and can vary considerably [11]–[13]. There is some evidence that eye color correlates with various psychological and biosocial factors, such as temperament [14]. Blue-eyed infants are reportedly more inhibited, shy, and timid than brown-eyed infants [14]–[16]. Similarly, Coplan et al. [17] found that boys with blue eyes were socially warier than boys with brown eyes, although such differences were absent between blue- and brown-eyed girls. In a previous study, we showed that eye color affects male face shape, specifically the mouth, eye, and chin area [18]. Brown-eyed males tend to have a face shape that suggests happiness and, hence, higher perceived trustworthiness while blue-eyed males tend to have a face shape that indicates anger and, hence, lower perceived trustworthiness [5], [18], [19]. There is also evidence that people are felt to be more honest if they are baby-faced with shorter chins and lower positioned features [20], [21]. Here we hypothesize that faces of brown-eyed individuals should be perceived as more trustworthy-looking than blue-eyed individuals because of certain stable facial features that correlate with eye color. Further, we wish to identify the specific features that evoke a perception of trustworthiness by using geometric morphometrics to analyze face shape variation among male and female university students.

## Methods

### Ethics Statement

The research was approved by The Institutional Review Board of Charles University, Faculty of Science. Written informed consent was obtained from all participants involved in our study. The data were analyzed anonymously.

### Photographs

The stimuli were photographs of 80 students (40 males: mean age = 20.8, range: 19–26, and 40 females: mean age = 21.2, range: 19–26) from the Faculty of Science, Charles University in Prague, Czech Republic. The students were seated in front of a white background and photographed by a digital camera, Canon 450D, using a studio electronic flash and a reflection screen. They were told to assume neutral, non-smiling expressions and to avoid any facial cosmetics and other facial ornaments. All photos were cropped so that the eyes were always horizontally at the same height with a standard length of neck visible. Pictures of individuals with either blue or brown eyes were selected. Individuals were excluded if they had intermediate eye colors, i.e., green.

### Rating of Photos

The photos were rated by two hundred and thirty eight participants (142 females and 98 males), mainly students of faculties other than the Faculty of Science, aged 23.1 years on average (females: mean age = 22.7, range: 19–48; males: mean age = 23.5, range: 18–48). Out of a total of 248 raters, 105 judged trustworthiness, 103 attractiveness, and 30 dominance. The raters differed in eye color: 99 had blue eyes (54 females, 45 males); 61 green eyes (38 females, 23 males); and 78 brown eyes (50 females, 28 males). Information about eye color was gathered by self-report. Everyone rated the whole set of 80 photos for trustworthiness (dominance/attractiveness) on a ten-point scale where 1 means very trustworthy and 10 very untrustworthy. All photos were presented and rated by means of the software application ImageRater 1.1.

Raters were recruited to judge the photos by email invitation. Each rater viewed them on a computer screen and judged them on a discontinuous ten-point scale. Each rater judged only one psychological factor. There was no time limit on the rating process. Photo order was randomized for each rating session. If a rater knew or was acquainted with the person on the photo, she/he was told not to rate it. For each rater, all ratings of all photos were converted to z-scores to eliminate the influence of individual differences among raters. Perceived trustworthiness, dominance, and attractiveness were calculated for each photo as its average z-score.

### Rating of Photos with Recolored Eyes

To find out whether it was eye color that affected perception of trustworthiness and not some other associated facial feature, we recolored the eyes on the facial photos from brown to blue and vice versa with Adobe Photoshop CS 3 software. This operation was performed in such a way that only the hue of the iris was changed, while the individually specific pattern of the iris remained intact.

The recolored photos were judged for perceived trustworthiness by a second group of 106 raters (35 males, 71 females) aged 21.4 years on average (males –22.0; females –20.7). Nobody who had rated the original set of photos was invited to judge the photos with recolored eyes. At the end of the rating session, the program asked whether the rater had noticed “something unusual” on the photos. No mention was made of the alteration to eye color.

### Facial Width-to-height Ratio

Photos of forty male students were measured by means of Image J software. Using the same methodology as in previous studies [7], [22], [23], we measured the distance between lip and brow (height of upper face) and between left and right cheekbones (bizygomatic width). Facial width-to-height ratio (WHR) was calculated as width divided by height. All distances were measured twice to improve measurement reliability. Reliability was high for all measures: distance between left and right cheekbones (r = 0.975, p<0.001); distance between upper lip and brow (r = 0.974, p<0.001); and width-to-height ratio (r = 0.961, p<0.001).

### Statistics

Individual trustworthiness, attractiveness, and dominance ratings were assessed for consistency using Cronbach’s alpha. The relationship between eye color and perceived trustworthiness was tested by *General Linear Models* (GLM) using a mean z-score of the trait as the dependent variable and eye color and sex of rated face as fixed factors. Because trustworthiness positively correlates with perceived attractiveness and negatively with perceived dominance, we included the ratings of these two factors in the model as covariates. Effect size was expressed by partial η^2^.

To test for interaction between rater’s eye color and photo’s eye color, we used *Linear Mixed Effect Models* (LME) available in SPSS 17 software. We used the model with a correlated residual within the random effects: facial photo identities were specified as a subject variable and rater identities as a repeated variable. Trustworthiness ratings were set as a dependent variable, with photo’s eye color and rater’s eye color as independent variables. The interaction “photo’s eye color * rater’s eye color” was specified in the model. Ratings of attractiveness and dominance were included in the model as covariates.

The differences in mean z-score between ratings of each photo before and after eye recoloring were calculated and tested separately for male and female raters by a one-sample t-test (H_0_: trustworthiness of original faces – trustworthiness of recolored faces = 0).

### Geometric Morphometrics

Photos of 40 men and 40 women (20 blue-eyed and 20 brown-eyed) were analyzed by geometric morphometric methods (GMM) in order (1) to find out which facial features were associated with a perception of trustworthiness and (2) to investigate the shape differences between blue-eyed and brown-eyed faces.

For this purpose, 72 landmarks (including 36 semilandmarks) were digitized using tpsDig2 software, ver. 2.14 [24]. Landmarks are points that are anatomically (or at least geometrically) homologous in different individuals, while semilandmarks denote curves and outlines. Landmark and semilandmark locations had been defined in previous studies [18], [25], [26]. Semilandmarks were situated by tpsRelw (ver. 1.49) software, using the following parameters: slide method = chord min BE, i.e., the curve would approximate the points with a minimum of deviation; maximum number of iterations set to 3; and option “slide recursive” left unchecked, i.e., the original configurations were used for every iteration). All configurations of landmarks and semilandmarks were superimposed using the generalized Procrustes analysis (GPA), available in tpsRelw, ver. 1.46. This procedure standardized the size of the objects and optimized their rotation and translation to minimize the distances between corresponding landmarks. To quantify variation among the landmark data configurations of all specimens, principal component analysis (PCA), i.e., relative warp analysis for parameter α = 0, was carried out using tpsRelw, ver. 1.46. [27]. To determine the face shape associated with perceived trustworthiness and eye color, we performed a multivariate regression with shape coordinates as the dependent variable and trustworthiness ratings or eye color as the independent variable, using tpsRegr, ver. 1.36 [28]. Shape regressions were displayed by thin-plate splines as deviations from the overall mean configuration (consensus) of landmarks.

To measure face shape variability by sex and by eye color, we chose variance, a common measure of morphological disparity (MD) [29], [30]. We used face shape data (partial warps including uniform component) to compute the variances for (1) males and females and for (2) blue-eyed and brown-eyed males. Then we used the multivariate analogue of Levene’s test (“betadisper” function within “vegan” package in R) to test for any significant difference in shape variability between groups [31]–[33]. In addition, we used body height and body mass index (BMI) to control for any potential allometric effect on face shape differences between blue-eyed and brown-eyed individuals. Because we could not access the raters’ BMIs, we used the BMI records of other students from the same population. A t-test was used to test the significance of body height and BMI differences between blue-eyed and brown-eyed males and females.

## Results

**Figure 1:**
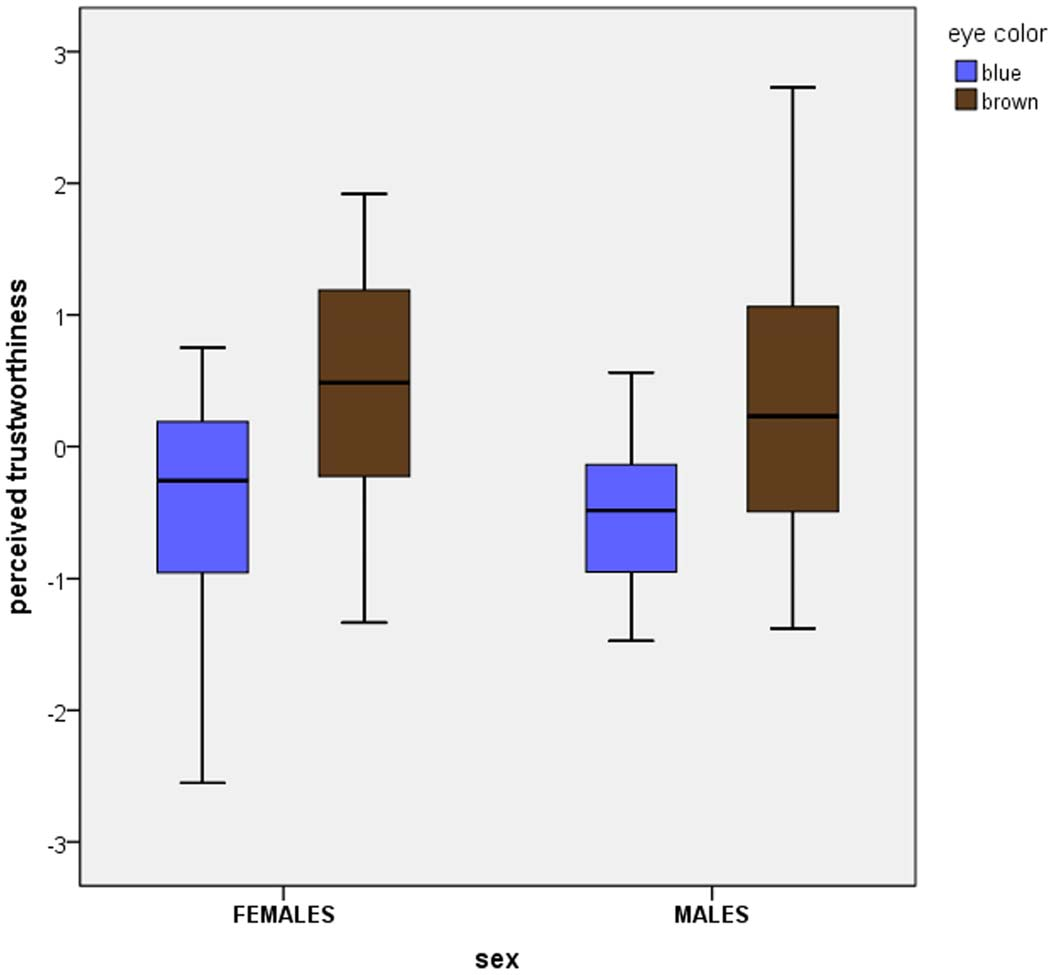
Perceived trustworthiness of blue-eyed and brown-eyed male faces and female faces. The y-axis shows residuals of perceived trustworthiness after statistical control for attractiveness and dominance (expressed by z-scores); whiskers denote standard deviations. Solid lines within the boxes indicate the group median.

Cronbach’s alpha values showed high inter-rater agreement for trustworthiness (0.970), dominance (0.835), and attractiveness (0.979). Eye color significantly influenced perceived trustworthiness (F_1,76_ = 4.24, p = 0.043, η^2^ = 0.053). The brown-eyed faces were perceived as more trustworthy than the blue-eyed ones. Sex of face had a significant effect on perceived trustworthiness (F_1,76_ = 59.90, p = 0.001, η^2^ = 0.441); female faces were rated as more trustworthy than male faces. We found a significant negative correlation between perceived trustworthiness and perceived dominance (r = −0.437, N = 80, p<0.01) and a positive correlation between perceived trustworthiness and perceived attractiveness (r = 0.829, N = 80, p<0.01). After we controlled for dominance and attractiveness, eye color still correlated with trustworthiness (F_1,74_ = 18.54, p<0.001, η^2^ = 0.2). Perceived dominance (F_1,74_ = 38.49, p<0.001, η^2^ = 0.342) and attractiveness (F_1,74_ = 126.82, p<0.001, η^2^ = 0.632) had a significant effect on perceived trustworthiness. Nevertheless, controlling for dominance and attractiveness did eliminate the effect of sex (F_1,73_ = 0.024, p = 0.877, η^2^ = 0.000), although the interactions “sex*dominance” (F_2,73_ = 19.21, p<0.001, η^2^ = 0.345) and “sex*attractiveness” (F_2,73_ = 68.28, p<0.001, η^2^ = 0.652) remained significant, indicating that male raters and female raters perceived attractiveness and dominance differently. For this reason we reran the analysis separately for male raters and female raters. Eye color had a significant effect on perception of trustworthiness by male raters (F_1,36_ = 6.72, p = 0.014, η^2^ = 0.157) and by female raters (F_1,36_ = 11.01, p = 0.002, η^2^ = 0.234). Both sexes considered the brown-eyed faces to be more trustworthy than the blue-eyed ones (Fig. 1).

We tested the possible effect of rater’s eye color on perceived trustworthiness with linear mixed-effects models. After we added rater’s eye color to the model, the relationship between photo’s eye color and perception of trustworthiness was significant for male faces (F_1,36_ = 6.882, p = 0.013) and for female faces (F_1,37_ = 13.721, p = 0.001). For male faces, rater's eye color had no effect on ratings of trustworthiness (F_2,__1327_ = 0.606, p = 0.546). For female faces, the effect was significant (F_2,1745_ = 3.928, p = 0.020). Blue-eyed female faces received lower ratings from brown-eyed raters than from blue- or green-eyed raters. Similarly, brown-eyed raters gave higher ratings to brown-eyed female faces than to blue-eyed female faces (F_1,74_ = 20.04, p<0.001). Despite some differences due to rater’s eye color, this factor was not primarily driving the ratings. All raters, irrespective of their own eye color, perceived the brown-eyed faces as more trustworthy than the blue-eyed ones.

We used a one-sample t-test to measure the effect of artificially recoloring the eyes, i.e., by comparing the mean change in trustworthiness of individual faces with a theoretical value of 0. Recoloring the eyes from blue to brown or from brown to blue did not systematically influence perceived trustworthiness (male faces: p = 0.818, t_39_ = 0.232, Mean±SE = 0.024±0.104; female faces: p = 0.748, t_39_ = −0.324, Mean±SE = 0.024±0.075). Thus, despite the higher attributed trustworthiness of brown-eyed faces, eye color itself had no significant effect on perceived trustworthiness.

Facial WHR negatively correlated with perceived trustworthiness of male faces (F_1,38_ = 5.197, p = 0.028, η^2^ = 0.12). Eye color was uncorrelated with facial WHR (F_1,38_ = 1.98, p = 0.168, η^2^ = 0.049), so this ratio did not significantly differ between blue-eyed male faces (Mean±SD = 1.895±0.127) and brown-eyed ones (Mean±SD = 1.949±0.107).

**Figure 2:**
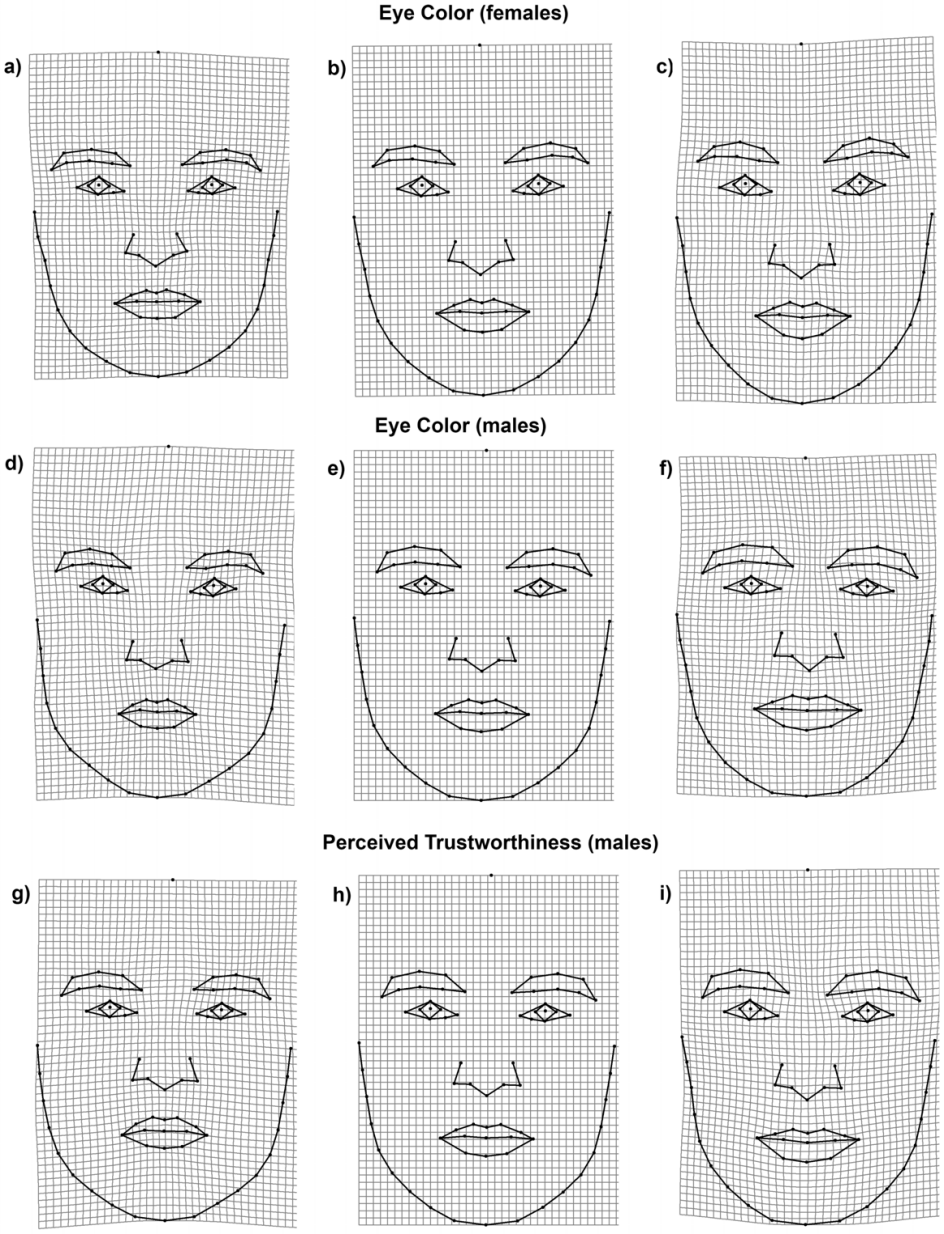
Shape changes associated with eye color and perceived trustworthiness. Thin-plate spline visualizations of the way face shape correlates with eye color (a–f) and trustworthiness (g–i). Generated face shapes of blue-eyed woman (a) and brown-eyed woman (c) compared to average female face (b). Generated face shapes of blue-eyed man (d) and brown-eyed man (f) compared to average male face (e). Generated face shapes of untrustworthy-looking man (g) and trustworthy-looking (i) man compared to average male face (h). The TPS grids of perceived trustworthiness for women are not shown because shape analysis did not meet statistical significance. The generated facial images (a–f) were magnified 3x for better readability.

Shape regression showed a significant correlation between perceived trustworthiness and male face shape (Goodall's F-test for 10,000 permutations: p = 0.0005, regression explained 8.6% of variance) but not for female face shape (p = 0. 17). Similarly, the correlation between eye color and face shape was significant only for male faces (Goodall's F-test for 10,000 permutations: p = 0.027, 5.5% of explained variance) but not for female faces (p = 0.058, 4.4% of explained variance) although the same trend was present. In contrast to blue-eyed male faces, brown-eyed male faces had a bigger mouth, a broader chin, a bigger nose, and more prominent eyebrows positioned closer to each other (Fig. 2d, 2f). A trustworthy male face was characterized by dilations of the TPS deformation grid in the mouth area and by constriction of the eyebrows (Fig. 2g, 2i). These characteristics, and the overall shape of a trustworthy face, matched TPS deformations in the direction of brown-eyed facial morphology. Female faces exhibited a similar pattern. Blue-eyed female faces tended to be oval with a less prominent and rounded chin, a mouth with corners parallel to the horizontal, and a longer distance between the eyes. In contrast, the narrower brown-eyed female faces tended to have a more prominent chin, a mouth with upward-pointing corners, and a shorter distance between the eyes (see Fig. 2a, 2c). The relationship between female face shape and perception of trustworthiness could not be reliably described because the correlation was not significant, nor did it show a trend.

Facial morphology was more variable in males than in females: p = 0.012, for 1000 permutations (male faces: MD = 0.00361, female faces: MD = 0.00285). Face shape variability did not significantly differ between brown-eyed male faces (MD = 0.00309) and blue-eyed ones (MD = 0.00373: p = 0.140, for 10,000 permutations). Body height did not significantly differ either between brown-eyed male faces and blue-eyed ones (t_38_ = −0.711, p = 0.481), nor did it differ by eye color among female faces (t_38_ = −0.312, p = 0.757). No difference in BMI was observed between blue-eyed and brown-eyed men (t_60_ = 0.219, p = 0.827) or between blue-eyed and brown-eyed women (t_188_ = 0.275, p = 0.783).

## Discussion

We found significant sex-dependent correlations between eye color, face shape, and perceived trustworthiness. Brown-eyed faces were perceived as more trustworthy than blue-eyed ones (See Fig. 1). There was also a relationship between eye color and face shape. Although the results were statistically significant only for male faces, the trend for female faces was in the same direction. The non-significant results for female faces might be due to lower phenotypic variability among women in general [34]. Blue-eyed male faces were characterized by a more angular and prominent lower face, a longer chin, a narrower mouth with downward-pointing corners, relatively smaller eyes, and rather distant eyebrows. This was also the pattern of an untrustworthy face. In contrast, brown-eyed faces tended to have a rounder and broader chin, a broader mouth with upward-pointing corners, relatively bigger eyes, and eyebrows closer to each other. This was also the pattern of a trustworthy face. Our findings are consistent with those of Todorov [5], i.e., when facial features associated with perceptions of higher (or lower) trustworthiness are exaggerated, the result is an expression of happiness (or anger) (See Fig. 2g–i). Further, the longer chin of blue-eyed individuals makes them look less baby-faced. It has been shown that higher ratings for honesty are given to more baby-faced people with shorter chins and lower-positioned facial features [20].

Given the negative correlation between perceived dominance and trustworthiness, our present results seemingly contradict the finding by Kleisner et al. [18] that brown-eyed men are perceived as being more dominant. It may be that eye color transmits two different signals. The perceived dominance of brown-eyed men may weaken but not totally eliminate the relationship between brown-eyed face shape and perceived trustworthiness. In support of the two-signal hypothesis, eye color had a stronger effect on trustworthiness when perceived dominance was controlled.

Our results lead to three questions. First, why would a man with a less robust face, characterized by a smaller nose, chin, and mouth, be perceived as less trustworthy? Second, why would these less robust faces be associated with blue eyes? And third, why would this association be stronger for male faces than for female faces?

Men with smaller facial features are perceived as less masculine, and therefore as less dependable and trustworthy. For instance, when Japanese women judge Japanese men, they tend to associate darker skin (itself a male characteristic) not only with greater masculinity, strength, and assertiveness, but also with manly sincerity and dependability [35]. Nevertheless, there is also evidence that more masculine faces are generally thought to be less cooperative, less honest, and less parenting-oriented [36]. Men with wider faces are perceived as less attractive and less trustworthy [7]. Keep in mind that facial WHR does correlate with various facial features that in turn correlate with perceived trustworthiness but not with eye color. The GMM analysis also showed that differences in perceived trustworthiness are not triggered solely by facial WHR. Therefore, other facial traits than facial WHR, such as the shape of the nose, the mouth, the chin, and the eye area, may primarily contribute to a perception of trustworthiness or untrustworthiness. The same facial features that make one seem more trustworthy, namely bigger eyes, larger eyebrows, a mouth with upward-pointing corners, and a generally extended and narrower face shape, are also perceived as being more attractive (see [37] for attractiveness visualization). To date, it seems that relatively smaller eyes and a smaller mouth with downward-pointing corners are the facial traits that cause the lower perceived trustworthiness of blue-eyed males.

Adaptation may also affect how one perceives the trustworthiness and attractiveness of a face [38]. The extent to which male (or female) faces are judged trustworthy (or untrustworthy) would thus depend on visual experience with a particular face shape. The first question might be answered conclusively by cross-cultural comparisons of attributes of masculinity/femininity and judgments of trustworthiness. To answer the second and third questions we propose two explanations.

### Ethnic Substructure

The first explanation is non-random association between alleles due to gene *linkage disequilibrium*. Eye color is mainly affected by one or more genes with large effect, and further modified by dozens of other genes [39]–[42]. Due to recombination, there can be no stable association between a particular eye color and a particular face shape. The current association of trustworthy-looking faces with brown eyes may be explained by genetic linkage disequilibrium, i.e., an immigrant population with a new phenotype has contributed to the gene pool in the recent past (less than several hundred years ago). Nevertheless, the blue-eyed and brown-eyed males in our study did not significantly differ in face shape variability. It thus seems improbable that a few individuals of mixed ethnic origin in one of the groups had caused the linkage between eye color and face shape, since their presence would have also increased that group’s face shape variability.

### Sex Linkage and Sexual Selection

The second explanation comes from the theory that hair and eye color diversified among early Europeans through intense sexual selection of women for novel and bright color traits [43]. Such selection should have produced some sex linkage, i.e., the new hair and eye colors should tend to appear more in women than in men, the proximal mechanism being sensitivity to estrogen during development. European-specific hair and eye colors, like blue eyes, would therefore correlate with other estrogen-influenced traits, such as facial structure. Some sex linkage does exist. A twin study has shown that hair is, on average, lighter-colored in women than in men, with red hair being especially more frequent, and that women show greater variation in hair color [44]. But why do blue eyes seem to correlate more strongly in men, than in women, with a more gracile facial structure? It may be that pre-natal exposure to estrogen is over-determined in women, i.e., all women are fully exposed to estrogen before birth regardless of their eye color. In men, the increase in pre-natal exposure would be confined to blue-eyed individuals.

Alternatively, a strong male preference for the new female features may have been accompanied by relaxed selection for other "pro-social" facial features due to the Hill-Robertson effect. The appearance of new facial features might have disrupted the existing evolutionary optimization of hormonal influences on different facial features. The faces of brown-eyed people would be seen as more trustworthy because they represent a biosocial adaptation that has been established for millions of years. Nevertheless, the blue-eyed phenotype must have provided its bearers with some kind of advantage to offset the loss of perceived trustworthiness.

Therefore, we tentatively suggest that a combination of sex linkage and sexual selection is the most probable explanation for the reported covariance between brown eyes and trustworthy-looking faces. Also, the blue-eyed phenotype is now abundant in Northern Europe and hence should have some kind of adaptive advantage, most likely one favored by sexual selection [43], [45], that compensates for the loss of perceived trustworthiness. The trade-off between a preference for colorful and visible physical features and the advantage of a trustworthy-looking face might have contributed to the high variability of European eye and hair color.

In conclusion, brown-eyed individuals tend to be perceived as more trustworthy than blue-eyed ones within a population with variable eye color, but it is not brown eyes that cause this perception. It is the facial morphology linked to brown eyes. Confirmation of this linkage will require analysis of the relationships between eye color, face shape, and perceived trustworthiness in other European countries, preferably in a population with as little ethnic substructure as possible. Such linkage is worth investigating, given the key importance of perceived trustworthiness in a broad range of social events from mate choice to business partner selection and to political marketing and democratic processes.
